# Stratified Impact of Therapies on Anaplastic Thyroid Cancer Outcomes in the Pre-Gene-Targeted Therapy Era

**DOI:** 10.1245/s10434-024-16852-y

**Published:** 2025-01-27

**Authors:** Kun Zhang, Xinyi Wang, Jianyong Lei, Anping Su, Tao Wei, Zhihui Li, Ya-Wen Chen

**Affiliations:** 1 Division of Thyroid Surgery, Department of General Surgery, West China Hospital, Sichuan University, Chengdu, Sichuan China; 2 Department of Otolaryngology, Icahn School of Medicine at Mount Sinai, New York, NY USA; 3 Department of Cell, Developmental, and Regenerative Biology, Icahn School of Medicine at Mount Sinai, New York, NY USA; 4 Black Family Stem Cell Institute, Icahn School of Medicine at Mount Sinai, New York, NY USA; 5 Institute for Airway Sciences, Icahn School of Medicine at Mount Sinai, New York, NY USA; 6 Center for Epithelial and Airway Biology and Regeneration, Icahn School of Medicine at Mount Sinai, New York, NY USA

**Keywords:** Anaplastic thyroid carcinoma, Response to treatments, Cohort study, Prognosis, Stratified outcomes

## Abstract

**Background:**

Anaplastic thyroid cancer (ATC) is a highly lethal disease, often diagnosed with advanced locoregional and distant metastases, resulting in a median survival of just 3–5 months. This study determines the stratified effectiveness of baseline treatments in all combinations, enabling precise prognoses prediction and establishing benchmarks for advanced therapeutic options.

**Methods:**

The study extracted a cohort of pathologically confirmed ATC patients from the Surveillance, Epidemiology, and End Results program. Overall, 1879 patients from 2000 to 2018 were identified from the database. Kaplan–Meier survival curve estimation and Cox proportional hazard regression were applied.

**Results:**

Overall, compared with no treatment, surgery raised 1-year overall survival (OS) from 0.6% to 30% and median survival from <1 month to 3 months in ATC patients. For stage IVa, surgery increased 1-year OS from 21.5% to 71.8% and median survival from 2 to 23.5 months, and in stage IVb, surgery increased 1-year OS from 9.4% to 41.3% and median survival from 2 to 7 months; however, in stage IVc, the benefits of surgery were not markedly different from non-surgical approaches. When combined with surgery, other effective non-surgical ATC treatments demonstrated a surgery-dominant synergistic effect, particularly for stages IVa and IVb ATC, but not for stage IVc ATC.

**Conclusions:**

Our study provides insights into stratified baseline treatments for patients with ATC in all stages, emphasizing surgery’s vital role in a multimodal approach.

**Supplementary Information:**

The online version contains supplementary material available at 10.1245/s10434-024-16852-y.

Anaplastic thyroid cancer (ATC), characterized by rapid progression and high mortality, presents significant treatment challenges.^1,2^ At diagnosis, 15–50% of patients with ATC present with locoregional invasion and distant metastases.^3,4^ Current therapies, albeit aggressive, yield a median overall survival (OS) of merely 3–5 months and a 20% 1-year survival rate, underscoring an urgent need for more effective interventions.^1,2,5^ While complete resection is the mainstay of therapeutic approaches for differentiated thyroid cancer (DTC), it is rarely suitable for patients with ATC at diagnosis^4,6,7^ due to the extent of disease and the unfavorable prognosis of incomplete palliative resection.^8^

With regard to therapeutic response for patients with ATC, most published reviews, series, and guidelines from the most updated European Society for Medical Oncology (ESMO, 2019)^4^ and American Thyroid Association (ATA, 2021) guidelines^3^ all advocate aggressive multimodality therapy in the absence of distant metastases. However, there is a lack of substantial backing evidence for these aggressive interventions, and estimates of their effectiveness remain inaccurate.^5,9^ On the other hand, targeted therapy represents a promising avenue in the treatment of patients with ATC, as it aims to exploit specific molecular abnormalities present within the tumor cells. Targeted inhibitors have shown the best efficacy thus far in a phase II clinical trial^10,11^ for BRAF-mutated patients with ATC. These targeted inhibitors include dabrafenib and trametinib (D+T), other tyrosine kinase inhibitors (TKIs)^12,13^ and anti-programmed death-1 (PD-1) antibody immune checkpoint inhibitors.^14,15^

Although targeted therapies have demonstrated potential, their integration into standard treatment algorithms has been slow, hindered by the lack of robust clinical trials and the pressing need for individualized treatment strategies.^16^ The absence of significant enhancement in OS rates for patients with ATC has necessitated a critical reassessment of baseline treatment approaches, which have been largely empirical, with limited evidence-based guidelines due to the disease’s rapid progression and the challenges inherent in conducting randomized controlled trials.^17^

The objective of this study was to categorize the therapeutic responses of patients with ATC to baseline treatment modalities, including surgery, chemotherapy, external beam radiation therapy (EBRT), and their combinations. By establishing a framework for understanding the response of patients with ATC to baseline treatment regimens, we seek to (1) provide evidence to support the practice of precision medicine in patients with ATC; and (2) build a comparison baseline for the efficacy of novel targeting and immune therapies in the future.

## Methods

### Data Sources

The study extracted a cohort of pathological confirmed patients with ATC from the Surveillance, Epidemiology, and End Results (SEER) program, a public database devoted to providing information on patient demographics, tumor morphology, stage at diagnosis, primary tumor site, first course of treatment, and follow-up for vital status and causes of death, in an effort to reduce the cancer burden among the US population.^18^ Given that the SEER database consists of de-identified patient information available to the public, our study did not require the approval of an Institutional Review Board. In conducting our research, we adhered to the Strengthening the Reporting of Observation Studies in Epidemiology (STROBE) guidelines for reporting observational data.^19^ In accordance with our previous studies,^20,21^ the selected database is cited as ‘Incidence – SEER Research Plus Data, 18 Registries, Nov 2020 Sub (2000–2018) – Linked To County Attributes – Total U.S., 1969-2019 Counties, National Cancer Institute, DCCPS, Surveillance Research Program, released April 2021, based on the November 2020 submission’.

### Study Cohort

The study cohort initially comprised 2504 consecutive patients with ATC who were pathologically diagnosed between 2000 and 2018, as selected retrospectively from the SEER registry. We excluded several groups of patients from our study: 244 who underwent palliative or exploratory surgeries without removal of the primary lesion; 180 who had a partial thyroidectomy or lobectomy; 198 who had confounding records of therapeutic radioactive iodine (RAI) treatment; and 7 for whom no survival time was documented. Ultimately, our final study cohort consisted of 1879 patients with ATC, all of whom either underwent the complete cancer removal procedure, total thyroidectomy (TT; defined in this study as operable patients), or did not receive any surgery (defined in this study as inoperable patients). The clinical data regarding demographics, tumor staging, and therapeutic approaches, i.e. age, sex, race, primary site of tumor, pathology, American Joint Committee on Cancer (AJCC) stage, primary surgery, radiotherapy, chemotherapy, survival months, and cause of death, were extracted.

### Outcome Definition

The cause of death was recorded to define whether each patient died from ATC, from causes unrelated to patients with ATC, or if they were alive at the end of the follow-up period. In our study, the primary outcome was OS, defined as the time from initial diagnosis to death from any cause or alive at the endpoint of the study.

### Survival Analysis

Univariate Cox proportional hazards regression and Kaplan–Meier curves were utilized to identify prognostic factors. Factors deemed significant by univariate analysis (*p* < 0.05) were subsequently included in the multivariate Cox proportional hazards models. The optimal Cox regression model was chosen through a backward selection process, with an entry criterion of *p* < 0.05 and an elimination criterion of *p* > 0.10. Multivariate Cox proportional hazards regression analyses were carried out to pinpoint variables that significantly impacted the OS of patients with ATC.

### Statistical Analysis

We have presented descriptive statistics in Table 1 for the entire study cohort and compared outcomes between patients with ATC who did or did not undergo TT. Continuous variables were analyzed using the Kruskal–Wallis test and expressed as mean ± standard deviation (SD)/median, while categorical variables were assessed using the Pearson Chi-square test and presented as number (percentage). All statistical analyses were performed using R Studio version 4.0.4. Kaplan–Meier curves were generated using GraphPad Prism 8.4.3 (GraphPad Software, Inc., San Diego, CA, USA). A two-tailed *p*-value of <0.05 was considered to indicate statistical significance.

**Table 1:** Baseline characteristics of patients with ATC with or without total thyroidectomy

	All	TT	No surgery	*p*-Value
*N*	1879	792	1087	
Age, years [mean ± SD/median]	69.3 ± 12.6/71.0	66.4 ± 13.3/68.0	71.3 ± 11.7/73.0	<0.001
Sex				0.645
Female	1136 (60.5)	474 (59.8)	662 (60.9)	
Male	743 (39.5)	318 (40.2)	425 (39.1)	
Ethnicity				0.069
White	1509 (80.3)	650 (82.1)	859 (79.0)	
Black	147 (7.8)	64 (8.1)	83 (7.6)	
Others^a^	223 (11.9)	78 (9.8)	145 (13.3)	
AJCC stage				< 0.001
IVa	160 (8.5)	96 (12.1)	64 (5.9)	
IVb	757 (40.3)	367 (46.3)	390 (35.9)	
IVc	332 (17.7)	96 (12.1)	236 (21.7)	
Unstaged	630 (33.5)	233 (29.4)	397 (36.5)	
AJCC N				< 0.001
N0	224 (11.9)	112 (14.1)	112 (10.3)	
N1a	83 (4.4)	48 (6.1)	35 (3.2)	
N1b	317 (16.9)	113 (14.3)	204 (18.8)	
Nx	1255 (66.8)	519 (65.5)	736 (67.7)	
AJCC M				< 0.001
M0	360 (19.2)	184 (23.2)	176 (16.2)	
M1	332 (17.7)	96 (12.1)	236 (21.7)	
Unspecified	1187 (63.2)	512 (64.6)	675 (62.1)	
Tumor size, cm				< 0.001
≤1	15 (0.8)	11 (1.4)	4 (0.4)	
>1 and ≤2	43 (2.3)	38 (4.8)	5 (0.5)	
>2 and ≤3	59 (3.1)	38 (4.8)	21 (1.9)	
>3 and ≤4	103 (5.5)	64 (8.1)	39 (3.6)	
>4 and ≤5	140 (7.5)	72 (9.1)	68 (6.3)	
>5	639 (34.0)	275 (34.7)	364 (33.5)	
Unspecified	880 (46.8)	294 (37.1)	586 (53.9)	
Tumor extension				< 0.001
Within thyroid capsule	200 (10.6)	100 (12.6)	100 (9.2)	
T3b	108 (5.7)	76 (9.6)	32 (2.9)	
T4a	464 (24.7)	250 (31.6)	214 (19.7)	
T4b	371 (19.7)	119 (15.0)	252 (23.2)	
Unspecified	736 (39.2)	247 (31.2)	489 (45.0)	
Radiotherapy				< 0.001
No	810 (43.1)	295 (37.2)	515 (47.4)	
EBRT	1069 (56.9)	497 (62.8)	572 (52.6)	
Chemotherapy				0.001
No	1134 (60.4)	444 (56.1)	690 (63.5)	
Yes	745 (39.6)	348 (43.9)	397 (36.5)	
Cause of deaths				< 0.001
Alive	171 (9.1)	149 (18.8)	22 (2.0)	
ATC	1441 (76.7)	538 (67.9)	903 (83.1)	
Other causes	267 (14.2)	105 (13.3)	162 (14.9)	
Survival, months [mean ± SD/median]	14.5 ± 34.8/3.0	27.2 ± 46.7/7.0	5.3 ± 17.4/2.0	< 0.001

Data are expressed as *n* (%) unless otherwise specified

*ATC* anaplastic thyroid cancer, *TT* total thyroidectomy, *SD* standard deviation, *AJCC* American Joint Committee on Cancer, *EBRT* external beam radiation therapy

^a^American Indian/Alaska Native, Asian/Pacific Islander

## Results

**Fig. 1:**
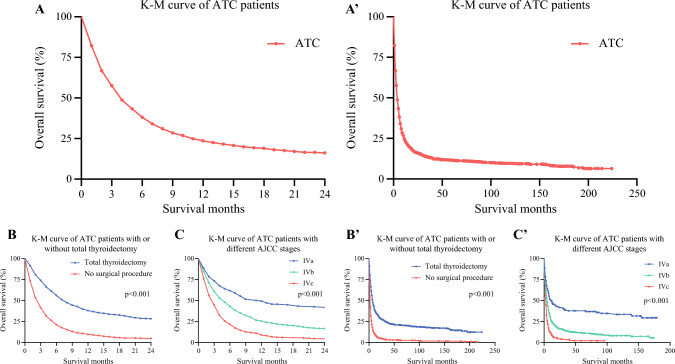
Kaplan–Meier curves illustrating the OS of patients with ATC. (**A**) OS in the short term (24 months); (**A′**) OS in the long term; (**B**) survival curves estimating OS in patients with ATC, with or without total thyroidectomy, in the short term (24 months); (**B′**) survival curves estimating OS in patients with ATC, with or without total thyroidectomy, in the long term; (**C**) survival curves estimating OS in patients with ATC across AJCC stages in the short term (24 months); (**C′**) survival curves estimating OS in patients with ATC across AJCC stages in the long term. *OS* overall survival, *ATC* anaplastic thyroid carcinoma, *K-M* Kaplan–Meier, *AJCC* American Joint Committee on Cancer

In this study, using the SEER database, we identified a cohort of 1879 patients with pathologically diagnosed ATC, spanning the period from 2000 to 2018. The characteristics of this population of patients with ATC included a diagnosis at an older age (median age of 71 years [range 16–85 years]), a predominance of White individuals (1509 patients, 80.3%), and a slight female preponderance, with a male-to-female sex ratio of approximately 2:3. The median follow-up duration for the study cohort was 3 months (ranging from 1 month to 224 months). During the follow-up period, there were 1708 deaths (90.9%), with 1441 (76.7%) attributed to patients with ATC and 267 (14.2%) attributed to non-thyroid causes, leading to cancer-specific death (CSS) as the primary cause. The 1-year CSS and OS rates for patients with ATC were 24.2% (95% confidence interval [CI] 22.2–26.3%) and 19.3% (95% CI 17.6–21.1%), respectively. The median survival time among patients with ATC was 3 months. Kaplan–Meier curves estimating OS for patients with ATC are illustrated in Figs. 1a and 1a′.

The study cohort was divided into two groups, based on whether patients had undergone complete thyroid removal surgery or not. The two groups were approximately equal in size, however there were noticeable disparities in clinical-oncological characteristics between the groups, except for sex (*p *= 0.645) ratio and ethnicity proportion (*p *= 0.069). The subcohort of operable patients was slightly younger, with a median age of 68 years, compared with 73 years in the inoperable subcohort. In addition, the TT subcohort had a higher proportion of patients staged as IVa and IVb (12.1% vs. 5.9%, and 46.3% vs. 35.9%, respectively) and a greater prevalence of localized lesions (T1-T3a: 12.6% vs. 9.2%; T3b: 9.6% vs. 2.9%; T4a: 31.6% vs. 19.7%; T4b: 10.5% vs. 23.2%) compared with the subcohort who did not undergo surgery. The acceptance of adjuvant/palliative therapy was slightly more favorable in the operable subcohort compared with the inoperable subcohort. Specifically, a greater proportion of patients in the operable subcohort received radiotherapy (62.8% vs. 52.6%) and chemotherapy (43.9% vs. 36.5%) compared with the inoperable subcohort. After the follow-up period, a noteworthy proportion of patients with ATC in the operable subcohort had survived (18.8%), with a median survival time of 7 months; the 1-year OS rate for this group was 41.8% (95% CI 38.6–45.3%). Conversely, for those patients who were inoperable, only 2.0% had survived, with a median survival time of 2 months; the 1-year OS rate was 9.0% (95% CI 7.5–10.1%). These results demonstrate the substantial disparity in survival rates between patients eligible for surgery and those who were not. To provide a clear visual representation of the impact of surgical intervention and AJCC staging on survival, we plotted Kaplan–Meier curves (Fig. 1). Detailed descriptive data for the study cohort are presented in Table 1 and electronic supplementary material (ESM) Table 1. The median survival time and 1-year OS associated with different therapeutic combinations are shown in Table 2.

**Table 2:** Median survival time and 1-year survival probability for all therapy combinations in the ATC cohort

Therapy regimens	Median survival time	1-year OS	
	Months	Survival rate (%)	95% CI (%)
No treatment	<1	0.6	0.3–1.0
Surgery alone	3	30.0	25.3–35.6
EBRT alone	2	4.7	3.2–7.1
Chemotherapy alone	3	11.4	6.7–19.3
Surgery + EBRT	7	36.7	31.2–43.7
Surgery + chemotherapy	7	30.4	20.1–46.0
EBRT + chemotherapy	4	15.3	12.3–19.0
All combined	9	41.8	37.4–46.8

*ATC* anaplastic thyroid cancer, *CI* confidence interval, *EBRT* external beam radiation therapy, *OS* overall survival

**Fig. 2:**
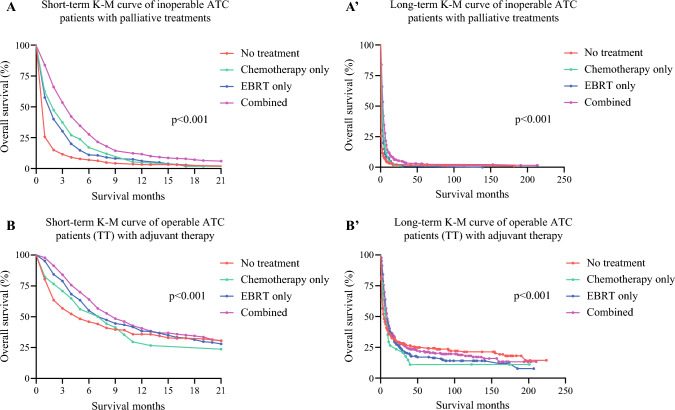
Survival curves showing OS outcomes of adjuvant/palliative therapies in patients with operable and inoperable ATC. (**A**) Short-term survival curves of inoperable patients with ATC who underwent palliative therapies; (**A′**) long-term survival curves of inoperable patients with ATC who underwent palliative therapies; (**B**) short-term survival curves of operable patients with ATC who underwent adjuvant therapies; (**B′**) long-term survival curves of operable patients with ATC who underwent adjuvant therapies. *OS* overall survival, *ATC* anaplastic thyroid carcinoma, *EBRT* external beam radiation therapy, *K-M* Kaplan–Meier

To further determine the potential survival benefit of radiotherapy, chemotherapy, and their combination in both the operable and inoperable subcohorts, we conducted two sets of Cox proportional hazard regression analyses. In the multivariate Cox analysis estimating OS of the operable subcohort, several factors were identified as significant prognostic indicators. Older age, with each increase in 1 year, was associated with a higher hazard ratio (HR 1.04, 95% CI 1.03–1.04; *p *< 0.001). Additionally, higher AJCC stage, particularly stage IVb compared with stage IVa (HR 2.23, 95% CI 1.64–3.03; *p *< 0.001), AJCC N1b compared with N0 (HR 1.42, 95% CI 1.04–1.94; *p *= 0.028), and tumor size >5 cm compared with ≤1 cm (HR 2.31, 95% CI 1.01–5.28; *p *= 0.046) were all identified as significant adverse factors affecting survival after adjustment and model selection in the univariate Cox regression. Radiotherapy (vs. no radiotherapy; HR 0.62, 95% CI 0.50–0.77; *p *< 0.001), unlike chemotherapy (vs. no chemotherapy; HR 0.93, 95% CI 0.63–1.36; *p *= 0.703) was the only therapeutic factor predicting better OS for the subcohort of operable patients with ATC. In the univariate Cox regression analysis of the inoperable population, AJCC stage, tumor size, and lymph node involvement did not meet the entry criteria (*p *< 0.05). As a result, the palliative purpose of chemotherapy and radiotherapy for the inoperable population with ATC was estimated to be beneficial prognostic factors (e.g. chemotherapy alone vs. no chemotherapy; HR 0.53, 95% CI 0.40–0.70; *p *< 0.001) in the final multivariate Cox model. Survival curves of the survival benefit of radiotherapy, chemotherapy, and their combination in both the operable and inoperable subcohorts are shown in Fig. 2. Detailed survival HRs are presented in ESM Table 3.

**Table 3:** Multivariate Cox proportional hazard regression for analyses of the operable and inoperable sub-cohort ATC patients for overall survival

Sub-cohort	Operable			Inoperable		
	HR	95% CI	P value	HR	95% CI	P value
Age (year)	1.04	(1.03–1.04)	< 0.001	1.01	(1.00–1.01)	0.005
AJCC stage						
IVa	1	reference		1	reference	
IVb	2.23	(1.64–3.03)	< 0.001	1.69	(1.19–2.39)	0.003
IVc	4.29	(2.88–6.40)	< 0.001	2.05	(1.43–2.94)	<0.001
Unstaged	1.37	(0.93–2.01)	0.113	1.46	(1.03–2.05)	0.033
AJCC N						
N0	1	reference				
N1a	1.23	(0.83–1.81)	0.297			
N1b	1.42	(1.04–1.94)	0.028			
Unspecified	1.45	(1.10–1.91)	0.009			
Tumor size						
<=1cm	1	Reference				
>1cm and <=2cm	1.27	(0.51–3.16)	0.606			
>2cm and <=3cm	1.21	(0.48–3.01)	0.686			
>3cm and <=4cm	1.78	(0.75–4.20)	0.190			
>4cm and <=5cm	1.67	(0.71–3.94)	0.244			
>5cm	2.31	(1.01–5.28)	0.046			
Unspecified	3.27	(1.39–7.69)	0.007			
Therapy regimen						
No	1	Reference		1	Reference	
Chemotherapy alone	0.93	(0.63–1.36)	0.703	0.53	(0.40–0.70)	<0.001
EBRT alone	0.62	(0.50–0.77)	< 0.001	0.58	(0.50–0.68)	<0.001
Combined	0.60	(0.50–0.73)	< 0.001	0.40	(0.34–0.47)	<0.001

*TT*, total thyroidectomy; *ATC*, anaplastic thyroid cancer; *HR*, hazard ratio; *CI*, confidential interval; *EBRT*, external beam radiation therapy.

**Fig. 3:**
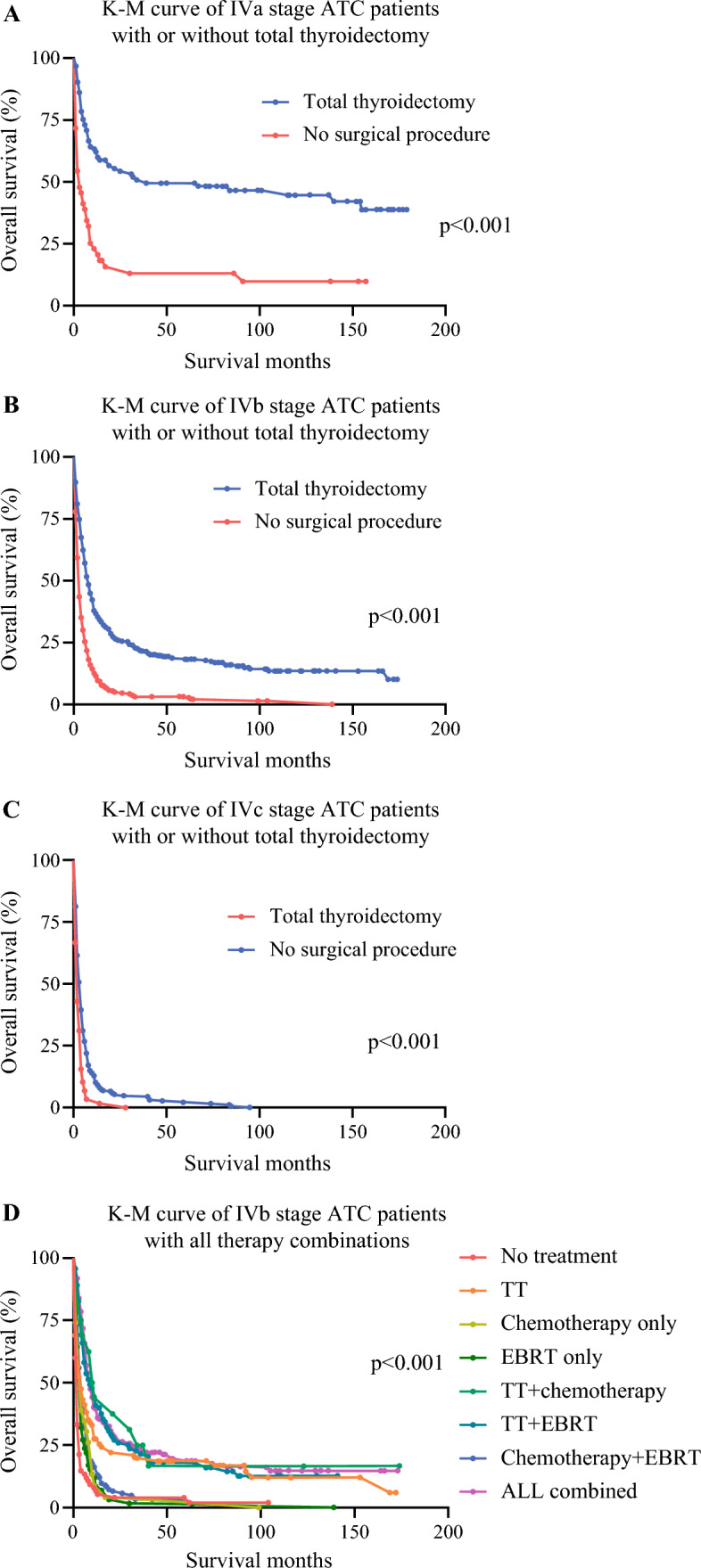
Impact of baseline therapy combinations on the overall survival outcomes for patients with stage-specific ATC. (**A**) Survival curves showing operable and inoperable patients with ATC at AJCC stage IVa; (**B**) survival curves showing operable and inoperable patients with ATC at AJCC stage IVb; (**C**) survival curves showing operable and inoperable patients with ATC at AJCC stage IVc; (**D**) survival curves showing patients with ATC who underwent all baseline therapy combinations at AJCC stage IVb. *ATC* anaplastic thyroid carcinoma, *AJCC* American Joint Committee on Cancer, *K-M* Kaplan–Meier, *TT* total thyroidectomy, *EBRT* external beam radiation therapy

To further investigate the largest population within this cohort of patients with ATC, which comprises 757 patients (40.3% of the cohort, as detailed in ESM Table 1), and to exclude patients with unspecified stage, we selected those with AJCC stage IVb as the secondary cohort for analysis. Stage IVb is also an important focus of our study because there is a lot of debate regarding the best approach for treating these patients. The baseline demographics and clinical-oncological characteristics remained consistent with those of the entire cohort of patients with ATC. Specific details are presented in ESM Table 2. Following adjustment through univariate regression analysis, another multivariate Cox regression was conducted. In this analysis, only age, tumor extension, and treatment combinations were identified as significant prognostic factors in the AJCC stage IVb population. All therapies, whether used alone or in combination, as illustrated in Fig. 3, possess distinct therapeutic value, with complete removal surgery having the most significant impact. ESM Table 4 presents the detailed data from the Cox regression model for patients with stage IVb ATC.

**Table 4:** Univariate and multivariate Cox proportional hazard regression for analyses of AJCC stage IVb sub-cohort ATC patients for overall survival.

	Univariate			Multivariate		
	HR	95% CI	*P* value	HR	95% CI	*P* value
Age (year)	1.03	(1.02–1.04)	< 0.001	1.02	(1.01–1.03)	<0.001
AJCC N						
N0	1	Reference				
N1a	0.77	(0.52–1.11)	0.163			
N1b	1.13	(0.86–1.48)	0.398			
Unspecified	1.40	(1.11–1.75)	0.004			
Tumor size						
<=1cm	1	Reference				
> 1cm and <=2cm	0.66	(0.19–2.31)	0.517			
> 2cm and <=3cm	0.93	(0.28–3.06)	0.906			
> 3cm and <=4cm	1.29	(0.40–4.13)	0.673			
> 4cm and <=5cm	1.12	(0.35–3.55)	0.848			
> 5cm	1.82	(0.58–5.68)	0.303			
Unspecified	2.73	(0.87–8.59)	0.086			
Tumor extension						
Within thyroid capsule	1	Reference		1	Reference	
T3b	1.21	(0.67–2.17)	0.533	1.41	(0.78–2.54)	0.262
T4a	1.59	(0.91–2.76)	0.104	1.93	(1.09–3.41)	0.023
T4b	2.31	(1.32–4.05)	0.003	2.22	(1.25–3.93)	0.006
Unspecified	2.06	(1.11–3.82)	0.023	0.88	(0.46–1.68)	0.700
Therapy regimen						
No	1	Reference		1	Reference	
Surgery only	0.27	(0.21–0.35)	< 0.001	0.28	(0.21–0.37)	<0.001
Chemotherapy only	0.37	(0.24–0.57)	< 0.001	0.41	(0.26–0.65)	<0.001
EBRT only	0.54	(0.42–0.72)	< 0.001	0.41	(0.31–0.54)	<0.001
Surgery and chemotherapy	0.15	(0.07–0.27)	< 0.001	0.14	(0.08–0.26)	<0.001
Surgery and EBRT	0.18	(0.13–0.23)	< 0.001	0.15	(0.11–0.21)	<0.001
Chemotherapy and EBRT	0.35	(0.27–0.45)	< 0.001	0.33	(0.25–0.42)	<0.001
All combined	0.17	(0.13–0.21)	<0.001	0.15	(0.11–0.20)	<0.001

*TT*, total thyroidectomy; *ATC*, anaplastic thyroid cancer; *HR*, hazard ratio; *CI*, confidential interval; *EBRT*, external beam radiation therapy.

## Discussion

In oncology, ATC is challenging because of its rare, aggressive nature, hindering randomized controlled trials and robust research. Consequently, ATC treatments are often empirical. Despite these challenges, our study aimed to precisely predict the survival benefits of different therapies for patients with ATC.

The current study, leveraging the robust SEER database, provides critical insights into the representative baseline management of patients with ATC. As expected, all traditional treatments showed efficacy in enhancing OS in patients with ATC, compared with no treatment at all, as illustrated in Tables 2 and Figs. 2 and 3. A pivotal finding from our analysis is the significant improvement in survival outcomes associated with complete thyroidectomy in select patients with ATC (Fig. 3). This observation aligns with existing literature that supports aggressive surgical intervention in patients with ATC, but emphasizes its application in selected cases.^2–4,22–24^ In analyzing the treatment outcomes for patients with ATC, it is essential to discern the incremental benefit of combining EBRT and chemotherapy with surgery. While surgery alone marked a substantial 30% increase in 1-year OS, the addition of EBRT or chemotherapy showed only a marginal improvement in survival rates. Specifically, surgery combined with EBRT led to a 36.7% increase in 1-year OS, and surgery with chemotherapy resulted in a 30.4% increase (Table 2). This suggests that while the addition of EBRT or chemotherapy to surgical intervention does enhance patient outcomes, the extent of this enhancement is relatively modest compared with the significant impact of surgery on its own. In light of these findings, it becomes apparent that the primary driver in improving 1-year OS in patients with ATC is surgery, and the role of EBRT and chemotherapy, although contributory, is less pronounced in the context of combined therapy. Additionally, the combined use of EBRT and chemotherapy, which resulted in a 15.3% increase in 1-year OS, is notably less effective compared with surgery alone, which achieved a 30% improvement in 1-year OS. Another critical observation is that the combination of all three approaches yielded the highest increase in 1-year OS, achieving a 41.8% improvement. It reveals a layered hierarchy of effectiveness in improving 1-year OS.

It is well-established that the outcomes for patients with ATC are largely -sensitive, which is also supported by our study (Figs. 1 and 3). There may be concerns that the distribution of the study cohort across AJCC stages (IVa: 160; IVb: 757; IVc: 332; and unstaged: 630) may introduce bias in evaluating the prognostic response to various therapy modalities. To address this issue and minimize the confounding effects associated with various disease stages, we focus on distinct stages of the disease in our Discussion. In stage IVa patients with ATC, as illustrated in Fig. 3a, surgery was found to be crucially important. It increased the 1-year OS rate from 21.5% to 71.8% and extended the median survival time from 2 months to 23.5 months, compared with patients who did not undergo surgery, aligning with the ATA^3^ and EMSO^4^ guidelines. In the case of stage IVc patients with ATC, as shown in Fig. 3c, the disparity between survival curves for those who underwent surgery and those who did not was not pronounced. The implication is that for stage IVc patients with ATC, multimodal adjuvant therapy might be more favorable in terms of OS compared with surgery alone. Lastly, we focused on stage IVb patients. For this group in particular, there has been a lot of controversy and disagreement about the best treatment options, making stage IVb a critical focus for evaluating the impact of different approaches. The Kaplan–Meier curves in Fig. 3d display the outcomes of various therapy modalities for stage IVb patients with ATC, showing two significant divergences, similar to those in Fig. 3b. This latter figure illustrates the bifurcations observed in patients treated with or without TT. This confirms that surgery is fundamental in determining the OS in stage IVb patients with ATC. The final multivariate Cox regression analysis for OS in stage IVb patients with ATC indicated that the combination of surgery with either EBRT alone (HR 0.15, CI 0.11–0.21; *p* < 0.001), chemotherapy alone (HR 0.14, 95% CI 0.08–0.26; *p* < 0.001), or both (HR 0.15, 95% CI 0.11–0.20; *p* < 0.001), resulted in a similar and substantial decrease in overall mortality. This was followed by surgery alone (HR 0.28, 95% CI 0.21–0.37; *p* < 0.001), the combination of EBRT and chemotherapy (HR 0.33, 95% CI 0.25–0.42; *p* < 0.001), chemotherapy alone (HR 0.41, 95% CI 0.26–0.65; *p* < 0.001), and EBRT alone (HR 0.41, 95% CI 0.31–0.54; *p* < 0.001). These patterns underscore the multidimensional nature of effective treatment protocols for patients with ATC. The combination of all three modalities, i.e. surgery, EBRT, and chemotherapy, emerges as the most advantageous strategy, suggesting a synergistic effect that maximizes patient survival outcomes. However, it is important to note that surgery remains the cornerstone of traditional treatment. Surgery will continue to be our most powerful tool until the advent of molecular-based targeted therapies, which may have already begun redefining treatment approaches, both now and in the foreseeable future.

ATC is known for its aggressive behavior and poor prognosis, and recent advances in genomic profiling have identified several genetic mutations that may play a pivotal role in its pathogenesis. One of the most commonly studied mutations is the BRAF mutation, which has been reported in approximately 20–50% of ATC cases.^11,25,26^ This mutation, along with others such as TP53 and RAS, presents potential opportunities for targeted therapy. Despite these important findings, it is important to acknowledge that the SEER database, used in this study, does not include detailed genetic mutation data. This limitation restricts our ability to directly assess the impact of such mutations on patient outcomes in this cohort. However, the results of some recent clinical studies have shown that the value of targeted therapy for ATC is promising.

In one study involving targeted therapies, MD Anderson Cancer Center^22^ compiled a comprehensive cohort of 479 patients treated over the past 2 decades. Among these patients, those who underwent surgery after receiving neoadjuvant BRAF-directed therapy (*n* = 20) experienced an impressive 1-year survival rate of 94%. This single-institution cohort study, which encompassed 479 patients with ATC spanning nearly 20 years, revealed significant increases in both 1- and 2-year survival rates. Specifically, 1- and 2-year survival rates rose from 35% and 18% in the 2000–2013 era (*n* = 227) to 47% and 25% in the 2014–2016 era (*n* = 100), and to 59% and 42% in the 2017–2019 era (*n* = 152), respectively. In another relatively large retrospective study (*n* = 104) led by the Memorial Sloan Kettering Cancer Center,^27^ the median survival for the overall cohort was 7 months, with a 1-year survival rate of 34%. Meanwhile, patients who were selectively treated with surgery and postoperative concurrent chemoradiation therapy (*n* = 53) exhibited a 1-year survival rate of 55%. These results, combined with insights from other smaller-scale studies,^1,28,29^ indicate that patients with ATC can be effectively managed with highly specialized, molecular-based personalized therapies. This includes the strategic use of surgery when it is considered suitable, regardless of the disease stage.

The limitations of our study are obvious. The retrospective design and dependence on the SEER database, while providing a broad dataset, introduces potential limitations. In this extracted cohort, we observed that a significant portion of patients in the SEER database lacked clear staging information, which posed certain challenges to our analysis. The missing staging information may lead to potential selection bias, affecting our assessment of patient prognosis. Another inherent bias is that patients who are able to undergo complete surgical resection for ATC are typically diagnosed at an earlier stage (IVa and IVb), which is associated with longer disease-free survival and OS. This creates a potential confounder, as the improved outcomes observed in these patients may be partially attributed to the earlier stage of their disease, rather than solely attributed to the therapeutic effects of surgery itself. Another significant limitation of our study is the absence of detailed information regarding the timing and use of targeted therapies for patients with ATC in the SEER database. Although targeted treatments for ATC were not as well-established between 2000 and 2018, this lack of information makes it challenging to build a comprehensive dataset in the SEER program. Acknowledging this limitation allows for a more nuanced interpretation of our findings and underscores the need for further research to investigate the role of targeted therapies in ATC as data become available.

## Conclusion

Our study offers significant insights into the stratified landscape of baseline treatments for patients with ATC, highlighting the critical role of surgery-centered multimodal therapy. This stratification not only improves our evidence-based understanding of current treatment outcomes but also establishes an essential benchmark for future therapeutic advancements. It is still uncertain whether aggressive local treatment in metastatic cases leads to improved survival or primarily enhances local disease control. Further investigation into this question, such as analyzing the cause of death in ATC patients who underwent TT and local treatments, using the National Cancer Database (NCDB) dataset, would provide valuable insights. Such research could help refine treatment guidelines and support more informed decision making for ATC patients with distant metastases.

## Supplementary Information

Below is the link to the electronic supplementary material.

Supplementary file1 (DOCX 26 KB)

Supplementary file2 (DOCX 21 KB)

## Data Availability

The data that support the findings of this study are available from each participating registry, but restrictions apply to the availability of these data, which were used under license for the current study and are therefore not publicly available.
