# Supplementary material for: Stratified Impact of Therapies on Anaplastic Thyroid Cancer Outcomes in the Pre-Gene-Targeted Therapy Era
Source: Ann Surg Oncol. 2025 Jan 27;32(4):2732–42. doi: 10.1245/s10434-024-16852-y (PMC11882711; doi:10.1245/s10434-024-16852-y)
Supplement: Supplementary file 1 — Supplementary file1 (DOCX 26 KB) [file 10434_2024_16852_MOESM1_ESM.docx]

Supplementary table 1. Baseline characteristics of ATC patients with different AJCC stages.

|  | **IVa** | **IVb** | **IVc** | **Unstaged** | **P value** |
| --- | --- | --- | --- | --- | --- |
| **N** | 160 | 757 | 332 | 630 |  |
| **Age (year), mean ± SD/median** | 68.2±13.7/71.5 | 69.4±12.6/71.0 | 67.7±12.2/68.0 | 70.1±12.6/73.0 | 0.010 |
| **Gender, n (%)** |  |  |  |  | 0.103 |
| Female | 97 (60.6%) | 480 (63.4%) | 185 (55.7%) | 374 (59.4%) |  |
| Male | 63 (39.4%) | 277 (36.6%) | 147 (44.3%) | 256 (40.6%) |  |
| **Ethnicity, n (%)** |  |  |  |  | 0.348 |
| White | 129 (80.6%) | 612 (80.8%) | 254 (76.5%) | 514 (81.6%) |  |
| Black | 12 (7.5%) | 65 (8.6%) | 27 (8.1%) | 43 (6.8%) |  |
| Others^a^ | 19 (11.9%) | 80 (10.6%) | 51 (15.4%) | 73 (11.6%) |  |
| **SEER stage, n (%)** |  |  |  |  | <0.001 |
| Localized | 107 (66.9%) | 12 (1.6%) | 0 (0.0%) | 0 (0.0%) |  |
| Regional | 22 (13.8%) | 266 (35.1%) | 0 (0.0%) | 69 (11.0%) |  |
| Distant | 31 (19.4%) | 454 (60.0%) | 332 (100.0%) | 204 (32.4%) |  |
| Unstaged | 0 (0.0%) | 25 (3.3%) | 0 (0.0%) | 357 (56.7%) |  |
| **AJCC N, n (%)** |  |  |  |  | <0.001 |
| N0 | 41 (25.6%) | 107 (14.1%) | 76 (22.9%) | 0 (0.0%) |  |
| N1a | 0 (0.0%) | 47 (6.2%) | 36 (10.8%) | 0 (0.0%) |  |
| N1b | 0 (0.0%) | 131 (17.3%) | 186 (56.0%) | 0 (0.0%) |  |
| Nx | 119 (74.4%) | 472 (62.4%) | 34 (10.2%) | 630 (100.0%) |  |
| **AJCC M, n (%)** |  |  |  |  | <0.001 |
| M0 | 45 (28.1%) | 315 (41.6%) | 0 (0.0%) | 0 (0.0%) |  |
| M1 | 0 (0.0%) | 0 (0.0%) | 332 (100.0%) | 0 (0.0%) |  |
| Unspecified | 115 (71.9%) | 442 (58.4%) | 0 (0.0%) | 630 (100.0%) |  |
| **Tumor size, n (%)** |  |  |  |  | <0.001 |
| <=1cm | 8 (5.0%) | 4 (0.5%) | 3 (0.9%) | 0 (0.0%) |  |
| >1cm and <=2cm | 14 (8.8%) | 22 (2.9%) | 5 (1.5%) | 2 (0.3%) |  |
| >2cm and <=3cm | 18 (11.2%) | 38 (5.0%) | 3 (0.9%) | 0 (0.0%) |  |
| >3cm and <=4cm | 20 (12.5%) | 57 (7.5%) | 26 (7.8%) | 0 (0.0%) |  |
| >4cm and <=5cm | 15 (9.4%) | 93 (12.3%) | 32 (9.6%) | 0 (0.0%) |  |
| >5cm | 51 (31.9%) | 399 (52.7%) | 189 (56.9%) | 0 (0.0%) |  |
| Unspecified | 34 (21.2%) | 144 (19.0%) | 74 (22.3%) | 628 (99.7%) |  |
| **Tumor extension, n (%)** |  |  |  |  | <0.001 |
| Within thyroid capsule | 146 (91.2%) | 18 (2.4%) | 36 (10.8%) | 0 (0.0%) |  |
| T3b | 0 (0.0%) | 93 (12.3%) | 15 (4.5%) | 0 (0.0%) |  |
| T4a | 0 (0.0%) | 352 (46.5%) | 112 (33.7%) | 0 (0.0%) |  |
| T4b | 0 (0.0%) | 248 (32.8%) | 123 (37.0%) | 0 (0.0%) |  |
| Unspecified | 14 (8.8%) | 46 (6.1%) | 46 (13.9%) | 630 (100.0%) |  |
| **Surgery, N (%)** |  |  |  |  | <0.001 |
| No surgery | 64 (40.0%) | 390 (51.5%) | 236 (71.1%) | 397 (63.0%) |  |
| TT | 96 (60.0%) | 367 (48.5%) | 96 (28.9%) | 233 (37.0%) |  |
| **Radiotherapy, N (%)** |  |  |  |  | 0.151 |
| No | 78 (48.8%) | 305 (40.3%) | 150 (45.2%) | 277 (44.0%) |  |
| EBRT | 82 (51.2%) | 452 (59.7%) | 182 (54.8%) | 353 (56.0%) |  |
| **Chemotherapy, N (%)** |  |  |  |  | 0.172 |
| No | 104 (65.0%) | 438 (57.9%) | 197 (59.3%) | 395 (62.7%) |  |
| Yes | 56 (35.0%) | 319 (42.1%) | 135 (40.7%) | 235 (37.3%) |  |
| **Cause of deaths, N (%)** |  |  |  |  | <0.001 |
| Alive | 49 (30.6%) | 66 (8.7%) | 10 (3.0%) | 46 (7.3%) |  |
| ATC | 79 (49.4%) | 588 (77.7%) | 282 (84.9%) | 492 (78.1%) |  |
| Other causes | 32 (20.0%) | 103 (13.6%) | 40 (12.0%) | 92 (14.6%) |  |
| **Survival months, mean ± SD/median** | 39.9±57.0/7.5 | 13.8±28.1/4.0 | 4.8±11.1/2.0 | 14.2±39.5/2.5 | <0.001 |

Abbreviations: ATC, anaplastic thyroid cancer; TT, total thyroidectomy; Others^a^, American Indian/Alaska Native, Asian/Pacific Islander; AJCC, American Joint Committee on Cancer; EBRT, external beam radiation therapy.
