# Supplementary material for: Stratified Impact of Therapies on Anaplastic Thyroid Cancer Outcomes in the Pre-Gene-Targeted Therapy Era
Source: Ann Surg Oncol. 2025 Jan 27;32(4):2732–42. doi: 10.1245/s10434-024-16852-y (PMC11882711; doi:10.1245/s10434-024-16852-y)
Supplement: Supplementary file 2 — Supplementary file2 (DOCX 21 KB) [file 10434_2024_16852_MOESM2_ESM.docx]

Supplementary Table 2. Baseline characteristics of AJCC stage IVb ATC patients with or without TT.

|  | **TT** | **No surgery** | **P value** |
| --- | --- | --- | --- |
| **N** | 367 | 390 |  |
| **Age (year), mean ± SD/median** | 67.0±12.8/69.0 | 71.7±11.9/74.0 | <0.001 |
| **Gender, n (%)** |  |  | 0.038 |
| Female | 219 (59.7%) | 261 (66.9%) |  |
| Male | 148 (40.3%) | 129 (33.1%) |  |
| **Ethnicity, n (%)** |  |  | 0.124 |
| White | 298 (81.2%) | 314 (80.5%) |  |
| Black | 37 (10.1%) | 28 (7.2%) |  |
| Others^a^ | 32 (8.7%) | 48 (12.3%) |  |
| **AJCC N, n (%)** |  |  | 0.003 |
| N0 | 60 (16.3%) | 47 (12.1%) |  |
| N1a | 33 (9.0%) | 14 (3.6%) |  |
| N1b | 61 (16.6%) | 70 (17.9%) |  |
| Nx | 213 (58.0%) | 259 (66.4%) |  |
| **Tumor size, n (%)** |  |  | <0.001 |
| <=1cm | 3 (0.8%) | 1 (0.3%) |  |
| >1cm and <=2cm | 20 (5.4%) | 2 (0.5%) |  |
| >2cm and <=3cm | 27 (7.4%) | 11 (2.8%) |  |
| >3cm and <=4cm | 37 (10.1%) | 20 (5.1%) |  |
| >4cm and <=5cm | 55 (15.0%) | 38 (9.7%) |  |
| >5cm | 187 (51.0%) | 212 (54.4%) |  |
| Unspecified | 38 (10.4%) | 106 (27.2%) |  |
| **Tumor extension, n (%)** |  |  | <0.001 |
| Within thyroid capsule | 10 (2.7%) | 8 (2.1%) |  |
| T3b | 65 (17.7%) | 28 (7.2%) |  |
| T4a | 204 (55.6%) | 148 (37.9%) |  |
| T4b | 86 (23.4%) | 162 (41.5%) |  |
| Unspecified | 2 (0.5%) | 44 (11.3%) |  |
| **Radiotherapy, N (%)** |  |  | 0.001 |
| No | 126 (34.3%) | 179 (45.9%) |  |
| EBRT | 241 (65.7%) | 211 (54.1%) |  |
| **Chemotherapy, N (%)** |  |  | 0.095 |
| No | 201 (54.8%) | 237 (60.8%) |  |
| Yes | 166 (45.2%) | 153 (39.2%) |  |
| **Cause of deaths, n (%)** |  |  | <0.001 |
| Alive | 60 (16.3%) | 6 (1.5%) |  |
| ATC | 258 (70.3%) | 330 (84.6%) |  |
| Other causes | 49 (13.4%) | 54 (13.8%) |  |
| **Survival months, mean ± SD/median** | 22.9±35.9/7.0 | 5.2±12.9/2.0 | <0.001 |

Abbreviations: ATC, anaplastic thyroid cancer; AJCC, American Joint Committee on Cancer; ATC, anaplastic thyroid cancer; TT, total thyroidectomy; Others^a^, American Indian/Alaska Native, Asian/Pacific Islander; EBRT, external beam radiation therapy.
